# Editorial: Brain injury and neurodegenerative diseases: imaging and mechanisms

**DOI:** 10.3389/fnmol.2024.1372246

**Published:** 2024-02-16

**Authors:** Zhuonan Wang, Iris Yuwen Zhou, Lei Gao, Li Chen

**Affiliations:** ^1^PET/CT Center, The First Affiliated Hospital of Xi'an Jiaotong University, Xi'an, China; ^2^Athinoula A. Martinos Center for Biomedical Imaging, Department of Radiology, Massachusetts General Hospital and Harvard Medical School, Charlestown, MA, United States; ^3^Department of Radiology, Zhongnan Hospital of Wuhan University, Wuhan, China; ^4^Department of Radiology, Affiliated Hospital of North Sichuan Medical College, Nanchong, China

**Keywords:** brain injury, MR imaging, cerebral small vessel disease, gangliosidosis, neurode generation

Brain injury is one of the most common acute and severe cases in neurosurgery, which is characterized by a high disability rate and high mortality rate. While brain injury may hasten the onset and advancement of neurodegenerative diseases in high-risk individuals, distinguishing the immediate effects of brain injury from those of a gradually unfolding neurodegenerative process, such as cognitive impairment, solely through clinical history and examination is challenging. The prognosis is uncertain, and viable treatment methods are limited.

Our Research Topic (*Brain injury and neurodegenerative diseases: imaging and mechanisms*) aims to explore the mechanism and imaging problems of related neuropathies with brain trauma and neurodegenerative diseases as disease models, to obtain guidance methods for early diagnosis and treatment of such diseases, achieve early clinical rehabilitation, obtain optimal prognosis and reduce the social and economic burden of patients.

A total of four articles were included in our Research Topics. For *Acute effects of single and repeated mild traumatic brain injury on levels of neurometabolites, lipids, and mitochondrial function in male rats* by Allen et al., published on June 29, 2023, researchers employed a male rat model of mild traumatic brain injury to overcome the constraints observed in prior studies that relied on a single traumatic blow. Utilizing a well-established awake-closed head injury paradigm to simulate mild traumatic brain injury, male rats underwent either a single injury or five injuries administered 1 day apart. The injuries were validated through a beam-walk task and a video observation protocol. The findings suggest that the decline in sensorimotor performance and the heightened presence of neurometabolic and lipidomic abnormalities are more pronounced after repeated, rather than singular, instances of mild traumatic brain injury. This underscores the cumulative impact of multiple concussions in quick succession. It emphasizes the need for additional preclinical research to unravel the underlying mechanisms behind these alterations, establish biomarkers, and inform treatment strategies aimed at enhancing patient outcomes.

*MR imaging and outcome in neonatal HIBD models are correlated with sex: the value of diffusion tensor MR imaging and diffusion kurtosis MR imaging* by Bao et al., published on September 15, 2023. Conducted using a full-term neonatal hypoxic-ischemic brain injury rat model, the research aims to assess the diagnostic and prognostic value of imaging techniques. The authors explored changes in diffusion kurtosis and diffusion tensor parameters in brain tissue over time, examining the development of complex behaviors and the impact of sex on these parameters. The results suggest a crucial complementary role of diffusion kurtosis and diffusion tensor parameters in the early identification of infants at risk for long-term harm, with sex exerting a significant influence. These findings hold promise for substantial and lasting improvements in the quality of life for newborns.

*Dynamic alterations in the amplitude of low-frequency fluctuation in patients with cerebral small vessel disease* by Song et al., published on September 22, 2023. This study uses dynamic amplitude of low-frequency fluctuation to investigate cerebral activity changes in cerebral small vessel disease (CSVD) patients. Unlike previous studies focusing on dynamic and static functional connections, this research emphasizes understanding the dynamic characteristics of local brain activity. Analysis revealed a broad spectrum of dynamic abnormalities in spontaneous brain activity among CSVD patients, particularly in regions related to memory, executive function, and emotion. The dynamic features in these brain regions correlate with neuropsychological scales, suggesting their potential as indices for evaluating CSVD symptoms.

*Characterization of a phenotypically severe animal model for human AB-Variant GM2 gangliosidosis* by Deschenes et al., published on November 22, 2023. This study on AB-Variant GM2 gangliosidosis (ABGM2) confirmed that the mild neurological phenotype in Gm2a–/– mice results from compensatory NEU3-mediated GM2 catabolism. Double knockout mice (Gm2a–/–Neu3–/–) emerge as a relevant model for severe ABGM2, offering insights into NEU3′s role in ganglioside catabolism and its impact on β-subunit expression in the brain. Unlike typical ABGM2 models, Gm2a knockout affects hexosaminidase isozyme activity. The Gm2a–/–Neu3–/– mice provide a more accurate representation of the disease, offering potential for assessing treatment effects on key symptoms and pathogenesis. These findings promise valuable insights for future research and potential treatments for ABGM2.

In future studies, we aim for imaging and mechanism investigations of various craniocerebral diseases, using brain injury and neurodegenerative diseases as models. This approach should be grounded in both basic and clinical research, incorporating interdisciplinary computing methods from engineering majors. The goal is to achieve breakthroughs and foster optimism in our research endeavors.

## Author contributions

ZW: Conceptualization, Funding acquisition, Investigation, Supervision, Writing—original draft, Writing—review & editing. IZ: Conceptualization, Investigation, Writing—original draft, Writing—review & editing. LG: Conceptualization, Investigation, Writing—original draft, Writing—review & editing. LC: Conceptualization, Investigation, Writing—original draft, Writing—review & editing.

